# Supplementary material for: Effect of Covid-19 on maternal and child health services utilization in Ghana. Evidence from the National Health Insurance Scheme (NHIS)
Source: PLoS One. 2024 Dec 26;19(12):e0311277. doi: 10.1371/journal.pone.0311277 (PMC11671015; doi:10.1371/journal.pone.0311277)
Supplement: S1 Appendix — (PDF) [file pone.0311277.s001.pdf]

APPENDIX

Table 1 *Number of facilities per type and ownership*

Facility Type	Mission	Private	Public	Total
CHPS Compound	7	0	57	64
Maternity Home	1	16	1	18
Clinic	1	38	0	39
Health Centre	123	2	57	182
Primary Care	80	35	67	182
Secondary Care	3	0	9	12
Tertiary Care	0	0	5	5
**Total**	**215**	**90**	**197**	**502**

Table 2 *Number of facilities per region and ownership*

Region	Mission	Private	Public	Total
AHAFO	6	1	1	8
ASHANTI	59	17	19	95
BONO	21	3	2	26
BONO EAST	4	2	3	9
CENTRAL	11	7	12	30
EASTERN	22	3	18	43
GREATER ACCRA	10	34	15	59
NORTH-EAST	4	0	3	7
NORTHERN	10	1	6	17
OTI	6	1	5	12
SAVANNAH	7	2	23	32
UPPER EAST	14	6	49	69
UPPER WEST	8	0	19	27
VOLTA	15	1	11	27
WESTERN	6	12	8	26
WESTERN NORTH	12	0	3	15
**Total**	**215**	**90**	**197**	**502**

Table 3 *Number of facility level per region*

Region	CHPS Compound	Clinic	Health Centre	Maternity Home	Primary Care	Secondary Care	Tertiary Care
AHAFO	0	0	4	0	4	0	0
ASHANTI	4	4	29	3	53	1	1
BONO	3	2	13	0	6	2	0
BONO EAST	0	1	1	0	7	0	0
CENTRAL	3	4	7	1	14	0	1
EASTERN	2	0	21	2	16	2	0
GREATER ACCRA	1	18	9	6	21	3	1
NORTH-EAST	1	0	2	0	4	0	0
NORTHERN	1	0	5	1	8	1	1
OTI	2	1	4	0	5	0	0
SAVANNAH	15	1	10	1	5	0	0
UPPER EAST	21	2	36	1	8	1	0
UPPER WEST	4	0	16	0	6	1	0
VOLTA	5	0	9	0	12	0	1
WESTERN	1	6	4	3	11	1	0
WESTERN NORTH	1	0	12	0	2	0	0
**Total**	**64**	**39**	**182**	**18**	**182**	**12**	**5**

Table 4 *Total hospital attendance by facility type/level*

Date	CHPS Compound	Clinic	Health Centre	Maternity Home	Primary Care	Secondary Care	Tertiary Care
Jan-18	16673	47818	123935	8004	472067	83987	40527
Feb-18	15383	47986	113002	6928	443261	75151	35743
Mar-18	14924	49811	112507	7347	483600	78348	34691
Apr-18	18434	45953	119139	7993	415357	78207	30738
May-18	18479	47413	120957	8082	447173	83138	28535
Jun-18	21757	47390	130781	8581	447839	80438	29420
Jul-18	21390	49556	138217	9317	475051	84995	26594
Aug-18	18293	44724	121407	7857	419934	82052	24700
Sep-18	17806	42588	114898	7234	407688	74612	25377
Oct-18	20971	50460	151114	8815	495563	91390	28569
Nov-18	20569	47887	144411	8552	471748	85867	28360
Dec-18	18396	43221	129904	7876	415792	72769	27110
Jan-19	6378	38743	70383	5627	405883	78298	24301
Feb-19	8883	39436	80641	6340	400375	80780	23085
Mar-19	9138	41684	88403	6494	424715	86067	23202
Apr-19	7186	40953	95802	5718	459625	89468	23614
May-19	10143	43783	118863	6797	502353	94891	25119
Jun-19	12970	44135	129148	6621	512791	90450	24289

Jul-19	15773	46059	146911	7430	576385	105274	27728
Aug-19	15388	41400	139126	6454	517305	93860	23712
Sep-19	14604	40113	141715	6466	491561	91189	23351
Oct-19	16249	50348	173184	7506	630709	112770	27937
Nov-19	13838	51447	163040	7557	598247	101072	24616
Dec-19	10214	40838	127167	6659	493959	89290	21999
Jan-20	9789	40140	123865	6638	518860	99639	24457
Feb-20	8888	39429	120372	7450	501650	94956	23453
Mar-20	8905	36812	109219	5540	464572	88641	21446
Apr-20	8555	25631	88482	4692	359583	54923	14797
May-20	10091	28281	90249	4763	355770	60316	15002
Jun-20	10728	31516	99507	5220	387677	71582	11514
Jul-20	10808	32096	114199	5816	406609	74207	17471
Aug-20	10141	30933	108670	5007	402526	75112	17162
Sep-20	9287	29371	98932	4817	387003	73248	15835
Oct-20	12497	34106	128156	6074	465022	89680	12253
Nov-20	11952	38549	130923	5467	606732	92607	18464
Dec-20	10513	36060	118875	5721	608945	91366	12114
Jan-21	8117	32063	106475	5132	561908	87533	15890
Feb-21	8401	30141	99303	5154	531632	81636	14947
Mar-21	9155	31388	103472	6127	563287	92440	15077
Apr-21	9482	29980	105486	7356	547137	79059	14289
May-21	10576	29796	107622	7233	554565	80641	18598
Jun-21	11040	32867	119883	7813	593789	90180	14421
Jul-21	12321	38160	137343	7980	629993	86112	15743
Aug-21	10976	33889	126894	7592	590469	82098	14117
Sep-21	11441	30940	126572	7000	605506	81773	13379
Oct-21	12492	32277	134431	7405	601769	79912	12517
Nov-21	11745	31643	125063	7153	593510	80827	14091
Dec-21	11055	34973	120766	8073	603652	59666	9841
**Total**	**612795**	**1874789**	**5739413**	**327481**	**23851148**	**4032516**	**1030194**

Table 5 *Average monthly hospital attendance across different health facility types/levels for the periods* ***precovid-19*** *and* ***covid-19****.*

	**CHPS Compound**	**Clinic**	**Health Centre**	**Maternity Home**	**Primary Care**	**Secondary Care**	**Tertiary Care**
PRECOVID-19	14,712	44743	124,573	7,321	478,057	87,652	26,970
COVID-19	10,467	32340	113,660	6,234	519,166	79,707	14,953
