# Supplementary material for: Effect of Covid-19 on maternal and child health services utilization in Ghana. Evidence from the National Health Insurance Scheme (NHIS)
Source: PLoS One. 2024 Dec 26;19(12):e0311277. doi: 10.1371/journal.pone.0311277 (PMC11671015; doi:10.1371/journal.pone.0311277)

APPENDIX

Fig 1 *Total utilization by specialty*

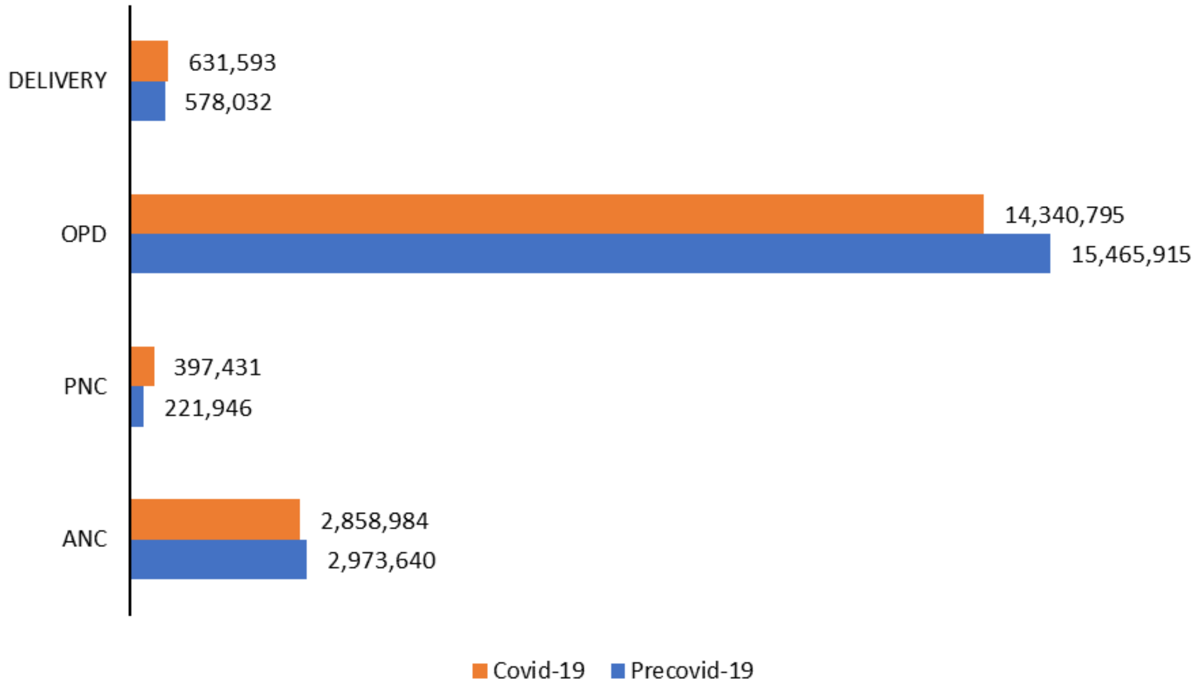

Fig 2 *Trend of Total utilization by specialty and facility type/level*

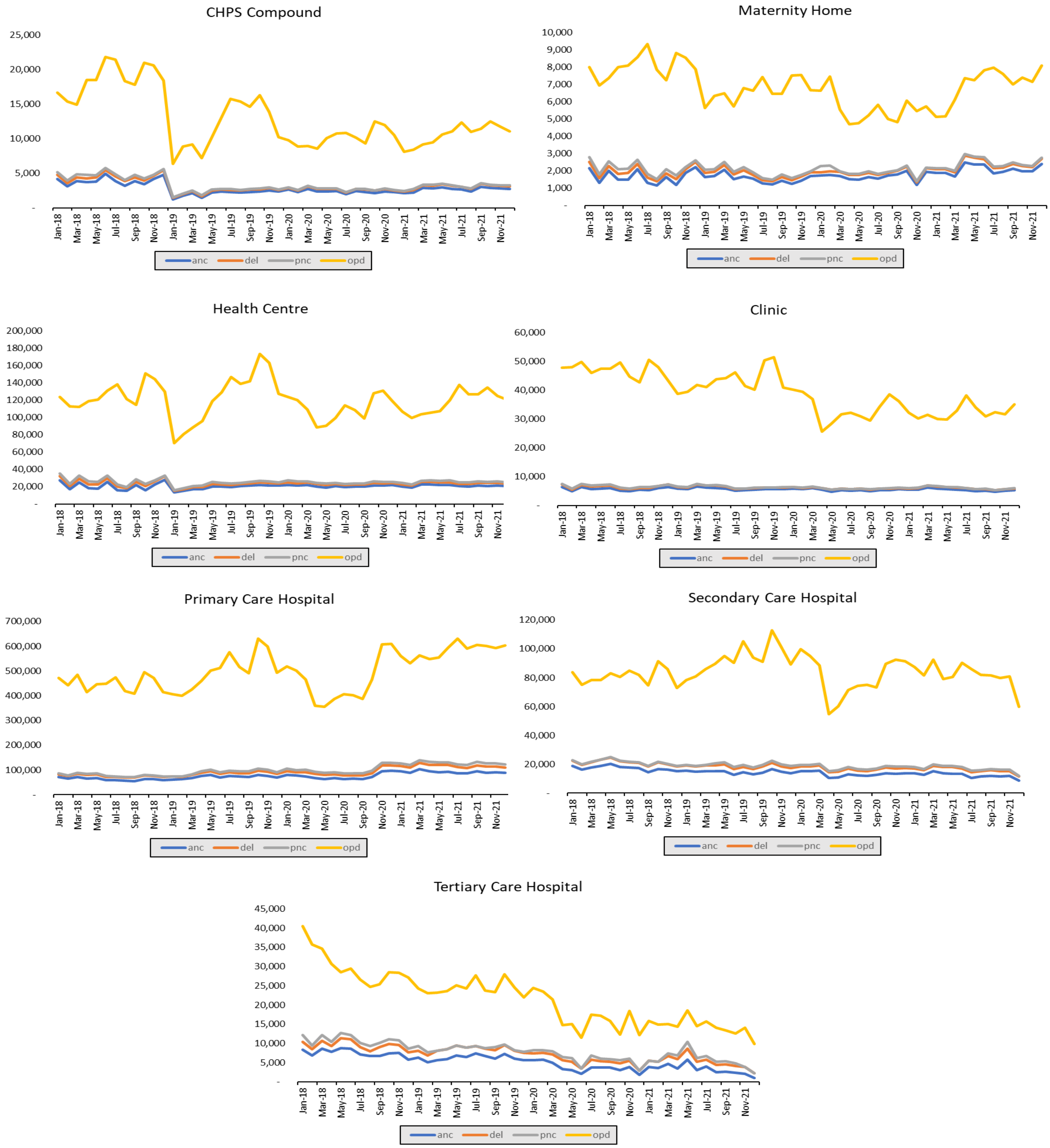

***Fig 3** Trend of Total utilization (**with precovid-19 and covid-19 periods**) by specialty and facility type/level*

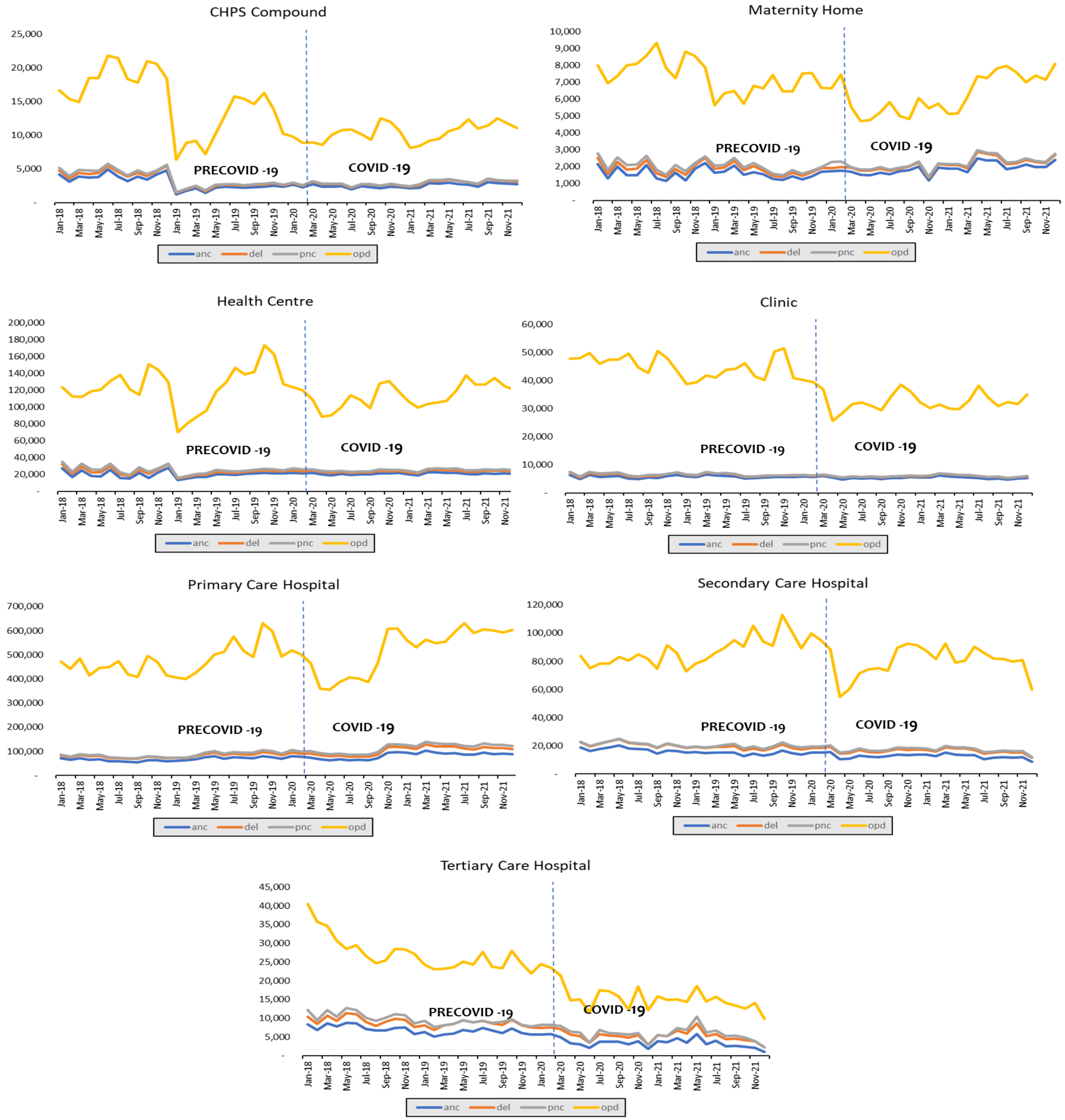

Supplement: S2 Appendix — (PDF) [file pone.0311277.s002.pdf]
