# Supplementary material for: Effect of Covid-19 on maternal and child health services utilization in Ghana. Evidence from the National Health Insurance Scheme (NHIS)
Source: PLoS One. 2024 Dec 26;19(12):e0311277. doi: 10.1371/journal.pone.0311277 (PMC11671015; doi:10.1371/journal.pone.0311277)
Supplement: S3 Appendix — (DOCX) [file pone.0311277.s003.docx]

*Total hospital attendance by facility type/level*
