# Supplementary material for: Effect of Covid-19 on maternal and child health services utilization in Ghana. Evidence from the National Health Insurance Scheme (NHIS)
Source: PLoS One. 2024 Dec 26;19(12):e0311277. doi: 10.1371/journal.pone.0311277 (PMC11671015; doi:10.1371/journal.pone.0311277)
Supplement: Attachment — Submitted filename: review_plosone.pdf [file pone.0311277.s004.pdf]

September, 30th, 2023.

Dear authors,

The manuscript “Effect of COVID-19 on maternal and child health services utilization in Ghana. Evidence from the National Health Insurance Scheme (NHIS)” submitted to PLOS ONE, addresses current and relevant topics for maternal and child health, in addition to presenting implications for the health system in the context investigated. However, the following points need to be reviewed:

1. The summary is adequate, concise and presents the central information of the study clearly, but I suggest that acronyms are not presented in this part of the text, that the type of sampling is described in the method and that the main results are expressed numerically.
2. On page 5, the acronym OPD is mentioned without its prior description in the main text. I recommend review.
3. The methodology appears to be adequate, however I recommend explaining the inclusion and exclusion criteria for the services that made up the study sample. Of the 37,468,336 consultations, what were the inclusion and exclusion criteria for those who made up the study sample?
4. In table 2 (page 10), why is the line referring to oct 2020 highlighted in bold? If the highlighting was intentional, it wasn't clear to me when reading why. I suggest review.
5. In table 3, page 12, line 224, the total average value has the decimal point in the incorrect position (78,0590.30). I recommend this correction and the review of the sum (780,590.31).
6. I recommend deepening the discussion on the tendency for an increase in services from the middle of 2021. Vaccination is timidly mentioned as a possible interfering variable in this change in the profile of services.
7. I recommend deepening the discussion, making the theoretical foundation used for comparison with the study findings more robust.

8. I identified an inconsistency between the number of citations 18 and 19, on page 20, respectively lines 352 and 357, with the references presented in the list of references. I recommend reviewing the numbering of references and citations.
9. In the appendix, table 3, page 28, line 560, is the title correct? Ownership information appears in the title although this content is not covered in the table. I suggest review.

Thank you for the opportunity to read your manuscript.
