# Supplementary material for: Effect of Covid-19 on maternal and child health services utilization in Ghana. Evidence from the National Health Insurance Scheme (NHIS)
Source: PLoS One. 2024 Dec 26;19(12):e0311277. doi: 10.1371/journal.pone.0311277 (PMC11671015; doi:10.1371/journal.pone.0311277)
Supplement: Attachment — Submitted filename: Response to Reviewer Comments.docx [file pone.0311277.s005.docx]

Dear Editor,

Thank you sincerely for your meticulous review and constructive feedback on our manuscript. Your insights have been invaluable in refining the quality of our work.

In response to your comments, we have diligently implemented necessary corrections throughout the manuscript. It is important to note that these revisions may have resulted in changes to line numbers and page numbers. Our commitment to enhancing the clarity and precision of our submission has been paramount in making these adjustments.

We genuinely appreciate your dedication to maintaining the academic rigor of our work, and your thoughtful suggestions have significantly contributed to its improvement.

Please find below our responses to the reviewers’ comments; the comments are italicized while the responses are highlighted in read. Moreover, the ensuing corrections or changes in the manuscript are highlighted yellow for easy recognition.

**Comment 1**: *The summary is adequate, concise and presents the central information of the study clearly, but I suggest that acronyms are not presented in this part of the text, that the type of sampling is described in the method and that the main results are expressed numerically*.

**Response 1**: The acronyms in the summary have been expounded, with due consideration given to their comprehensive presentation. Additionally, the method section now explicitly outlines the type of sampling employed, further enhancing clarity and transparency. The key finding of the results have also been presented in numerical values.

**Comment 2**: *On page 5, the acronym OPD is mentioned without its prior description in the main text. I recommend review*.

**Response 2**: The acronym OPD has been judiciously introduced into the main text, ensuring a seamless and coherent narrative.

**Comment 3**: *The methodology appears to be adequate, however I recommend explaining the inclusion and exclusion criteria for the services that made up the study sample. Of the 37,468,336 consultations, what were the inclusion and exclusion criteria for those who made up the study sample?*

**Response 3**: *A meticulous elucidation of the inclusion and exclusion criteria in the selection of services within the study sample has been incorporated into the methodology section, page 7, lines 145 - 155. This ensures a lucid understanding of the criteria shaping the dataset*.

**Comment 4**: *In table 2 (page 10), why is the line referring to oct 2020 highlighted in bold? If the highlighting was intentional, it wasn't clear to me when reading why. I suggest review.*

**Response 4**: The formatting anomaly in Table 2, specifically the bold highlighting of the line referring to October 2020, has been rectified to uphold precision and consistency in presentation.

**Comment 5**: *In table 3, page 12, line 224, the total average value has the decimal point in the incorrect position (78,0590.30). I recommend this correction and the review of the sum (780,590.31*).

**Response 5**: The decimal point in Table 3 has been diligently rectified, addressing the highlighted discrepancy and ensuring numerical accuracy in the presentation. The total average now reads 780,590.31

**Comment 6**: *I recommend deepening the discussion on the tendency for an increase in services from the middle of 2021. Vaccination is timidly mentioned as a possible interfering variable in this change in the profile of services*.

**Response 6**: The discussion on the discernible increase in services from mid-2021 has been substantially enriched (see page 12, lines 323 - 337. A comprehensive exploration of potential contributing factors, with a nuanced emphasis on the role of vaccination, has been incorporated, elevating the depth and scholarly discourse of the analysis.

**Comment 7**: *I recommend deepening the discussion, making the theoretical foundation used for comparison with the study findings more robust*.

**Response 7**: The discussion section has undergone a rigorous revision, bolstering its theoretical underpinnings, line 360 - 375, 385 - 404 and 421 - 429. A more robust integration of relevant theoretical frameworks has been undertaken to fortify the comparative analysis with the study's empirical findings.

**Comment 8**: *I identified an inconsistency between the number of citations 18 and 19, on page 20, respectively lines 352 and 357, with the references presented in the list of references. I recommend reviewing the numbering of references and citations*.

**Response 8**: A meticulous review and adjustment of reference and citation numbering have been conducted to align seamlessly, preserving scholarly precision and adherence to academic conventions. Specifically, the numbering of references and citations have been reviewed to 17 and 18 on pages 21 and 22, respectively lines 380 and 405. Additionally, three (3) new references have been introduced to augment the theoretical frameworks, numbering 19, 20 and 21.

**Comment 9**: *In the appendix, table 3, page 28, line 560, is the title correct? Ownership information appears in the title although this content is not covered in the table. I suggest review*.

**Response 9**: *The title of Table 3 in the appendix has been aptly rectified to accurately reflect the table's content for clarity and precision. The revised title now appropriately denotes “Number of facility level per region, page 31, line 637.*

We hope our revision will meet your expectations.

Yours faithfully

Edward Nketiah-Amponsah (On behalf of authors)
